# Self-reported drug use six months after a brief intervention: do changes in reported use vary by mental health status?

**DOI:** 10.1186/1940-0640-7-S1-A6

**Published:** 2012-10-09

**Authors:** Antionette Krupski, Jeanne Sears, Jutta Joesch, Sharon Estee, Lijian He, Alice Huber, Chris Dunn, Peter Roy-Byrne, Richard Ries

**Affiliations:** 1 Center for Healthcare Improvement for Addictions, Mental Illness, and Medically Vulnerable Populations (CHAMMP), Harborview Medical Center, University of Washington School of Medicine, Seattle, WA, USA; 2 Research and Data Analysis Division, Washington State Department of Social and Health Services, Olympia, WA, USA; 3 Division of Behavioral Health and Recovery, Washington State Department of Social and Health Services, Health and Recovery Services Administration, Olympia, WA, USA

## 

Although brief intervention (BI) for alcohol and other drug problems has been associated with decreased levels of self-reported substance use, there is little information in the literature as to whether individuals with co-occurring hazardous substance use and mental illness would benefit from BI to the same extent as those without mental illness, despite high estimates comorbidity. This study took advantage of a naturalistic situation where a screening, brief intervention, and referral to treatment (SBIRT) program had been implemented in a large hospital emergency department in Seattle, WA. A subset of patients who received BI was interviewed six months following the intervention about current alcohol/drug use. A search of the patients’ medical records allowed us to divide the sample into those with and without evidence of mental illness and to analyze their self-reported drug use. Although unadjusted means indicated a possible decrease in illegal drug use between baseline and follow-up interviews for both groups, there was no significant difference between mentally ill and non-mentally ill subgroups with regard to self-reported changes in substance use. Changes in self-reported alcohol and binge drinking days showed a similar pattern. Although the lack of a comparison group prevented us from attributing change in reported use to BI alone, these data suggest the impact of BI on subsequent drug and alcohol use may not differ among patients with mental-health diagnoses.

